# Prevalence of intestinal parasitic infection and its association with anemia among pregnant women in Wondo Genet district, Southern Ethiopia: a cross-sectional study

**DOI:** 10.1186/s12879-019-4135-8

**Published:** 2019-05-30

**Authors:** Amelo Bolka, Samson Gebremedhin

**Affiliations:** 1 Wonedo Genet Town Health Unit, Wondo Genet, Ethiopia; 2 Department of Public Health, St Paul’s Hospital Millennium Medical College, Addis Ababa, Ethiopia

**Keywords:** Intestinal parasitic infection, Intestinal helminths, Anemia, Pregnancy, Hemoglobin, Ethiopia

## Abstract

**Background:**

Previous studies reported contradicting findings about the association between intestinal parasitosis and maternal anemia. In this study we aimed to determine the prevalence of intestinal parasitic infection and its association with anemia among pregnant women in Wondo Genet district, Southern Ethiopia.

**Methods:**

This facility-based cross-sectional study was conducted in June and July 2018. Pregnant women (*n* = 352) were randomly drawn from five health centers using antenatal care follow-up lists. Trained data collectors administered the questionnaire. Capillary blood was collected and analyzed for hemoglobin using the HemoCue method. Stool sample was collected following standard procedure and analyzed for the presence and types of intestinal parasites using direct microscopy with Formalin-ether concentration technique. Association between intestinal parasitosis and anemia was measured using multivariable binary logistic regression analysis. The outputs are presented using adjusted odds ratio (AOR) with 95% confidence intervals (CI).

**Results:**

The overall prevalence of intestinal parasitic infection was 38.7% (95% CI: 33.6–43.8%). One-tenth (9.7%) of the pregnant women were infected with polyparasites. *Ascaris lumbricoides* was the predominant infection encountered in 24.9% of the women. The other infections identified were: hookworms (11.2%), *Giardia lamblia* (5.4%), *Entamoeba histolytica* (3.4%), *Trichuris trichiura* (2.9%) and *Schistosoma mansoni* (2.3%). The mean (± standard deviation) hemoglobin concentration was 12.3 (±1.9) g/dl and 31.5% (95% CI: 26.6–36.4%) women were anemic (hemoglobin < 11 g/dl). The prevalence of anemia among women infected with intestinal parasite (55.6%) was substantially higher than the prevalence in their counterparts (16.4%) (*p* <  0.001). In a multivariable model adjusted for multiple potential confounders including socio-economic status indicators, the odds of anemia were six times increased (AOR = 6.14, 95% CI: 2.04–18.45) among those affected by at least one intestinal parasite.

**Conclusion:**

Strengthening the existing water, sanitation and hygiene programs and routine deworming of pregnant mothers may help to reduce the burden of both intestinal parasitic infection and anemia in pregnant women.

**Electronic supplementary material:**

The online version of this article (10.1186/s12879-019-4135-8) contains supplementary material, which is available to authorized users.

## Background

Between 1990 and 2010, the global prevalence of anemia declined by nearly 20% from 40 to 33% [1]. Yet, anemia remains a major concern with moderate or severe public health significance in more than 80% of the world countries [2]. In 2010 anemia affected more than two billion people globally and contributed to 9% of total years of life lived with disability [1]. Children under five years of age, pregnant women and women of reproductive age bear the highest burden [1, 2]. Anemia prevalence exceeds 50% in many regions of the sub-Saharan Africa [1]. According to the 2005 estimate, anemia has severe public health significance in Ethiopia [2]. In 2016, the national prevalence among pregnant women was 29% and the figure did not decline over the previous decade [3, 4].

Anemia has multifaceted and frequently coexisting etiologies. Iron deficiency is regarded as the primary cause contributing to nearly 50% of the global burden of anemia [1, 2]. Parasitic infections including malaria, hookworms and schistosomiasis may explain nearly a quarter of anemia [1]. Further hemoglobinopathies, acute or chronic infections and other nutritional deficiencies have ethological significances [1, 2]. During pregnancy, hemodilution – disproportionate increase in plasma volume as compared with red cell mass – is a key cause of prenatal hemoglobin decline. Contextual factors including poverty and limited access to health care are also important root causes [1].

Maternal anemia leads to multiple and serious consequences. Observational studies suggested that anemia in pregnancy increases the risks of maternal mortality and low birthweight [5]. A systematic review confirmed that 1 g/dl increase in mean hemoglobin level in late pregnancy is associated with nearly 30% reduction in the odds of maternal mortality [6]. Further prenatal iron supplementation during pregnancy increases birthweight and reduces risk of prematurity [7–9]. Anemia in pregnancy may also increase risks of premature birth and perinatal mortality [10].

According to the World Health Organization (WHO), about one-fourth of the world population is infected with intestinal parasites primarily *Ascaris lumbricoides, Trichuris trichiura* and hookworms [11]. Intestinal parasites may cause or aggravate anemia via multiple and interactive mechanisms [12, 13]. Hook worms,schistosomiasis and*, Trichuris trichiura* lead to intestinal blood loss. Many intestinal parasites reduce appetite and compromise nutrient intake. Further helminthiasis-inducted intestinal inflammation may limit absorption of nutrients. Intestinal parasites may promote indigenous nutrient loss by inducing intestinal mucosa damage, impairing digestion and causing diarrhea. *Giardia lamblia and Entamoeba histolytica* also cause blood loss and inflammation-induced restriction of iron absorption [12, 13].

The association between intestinal parasitosis and anemia among children is well established and systematic reviews suggested deworming after a confirmed infection results in a significant hemoglobin rise in children [5, 13]. However, the significance of parasitic infection to anemia in pregnancy has not been thoroughly explored and the existing evidence is equivocal. Observational studies conducted in Hadiya district, Southern Ethiopia [14], Western Ethiopia [15], Nigeria [16], Venezuela [17] and Philippines [18] reported positive associations. Conversely, a study conducted in Papua New Guinea found no significant relationship [19]. Further, according to a systematic review, a single dose of antihelminthic in the second trimester of pregnancy showed no effect on the hemoglobin status of pregnant women [20].

In this cross-sectional study we aimed to determine the prevalence of intestinal parasitic infection and its relationship with anemia among pregnant women in Wondo Genet district, Southern Ethiopia.

## Methods

### Study design

This facility-based cross-sectional study was conducted in June and July 2018 among pregnant women booked for antenatal care (ANC) in five public health centers of Wondo Genet district, Southern Ethiopia.

### Study setting

Wondo Genet is situated in Sidama zone, Southern Ethiopia and has an estimated population size of 163,000. Administratively it is classified into 13 rural and 2 urban villages. The altitude of the district ranges from 1761 to 2695 m above sea level (ASL) and the area is characterized by two distinct agro-ecological zones. Midlands, land mass ranging between 1750 to 2300 m ASL, contribute for 62% of the population; whereas, highlands (above 2300 m ASL), contribute for the remaining 38% the population. The average rainfall is 1120 mm per annum and according to the season, temperatures vary from 7 to 26 °C. The main rainy season is between July and early October. About 85% of the population livelihood depends on subsistent farming. The district has 5 health centers and 16 functional health posts.

In Ethiopia the constellation of services provided within the ANC package include, but not limited to, iron folic acid supplementation, deworming, HIV testing, nutrition counseling and intermittent preventive treatment for malaria.

### Sample size determination and sampling approach

Sample size adequate for estimating prevalence of anemia and intestinal parasitosis was determined independently using single population proportion formula. The specifications made in the computation were: 95% confidence level, 5% margin of error, 32.8% expected prevalence of anemia [21], 29.5% expected prevalence of intestinal parasitosis [22] and 10% compensation for possible non-response. Ultimately sample size of 352 was judged to be sufficient.

Similarly, the adequacy of the sample size for determining the association between intestinal parasitosis and anemia status was evaluated using double population proportion formula using Epi InfoTM 7 statistical package with the inputs of: 95% confidence level, 80% power, 1:3 ratio between subjects with and without intestinal parasitosis and odds ratio (OR) of 2.4 [23].

Study participants were selected from the five health centers using stratified sampling approach (i.e. considering each health facility as stratum). During the survey 1739 women were booked for ANC and were eligible for the study. Initially, the total sample size was allocated to the facilities proportional to their ANC clients flow rate. Ultimately the individual women were selected using systematic random sampling technique with sampling interval of five.

### Data collection

Data were collected using pretested, structured and interviewer administered questionnaire by seven trained nurses and public health officers. Socio-demographic related questions were directly extracted from the standard Demographic and Health Survey (DHS) questionnaire [3]. Gestational age was determined based on women’s recall of last normal menstrual period. Knowledge of the mothers on anemia was assessed using a set of questions developed by the investigators. Dietary diversity of the women was assessed using the Women’s Dietary Diversity Score questionnaire of the Food and Agriculture Organization of the United Nations (FAO). The respondents were asked whether they ate from nine standard food groups in the previous day of the survey without setting minimum intake restrictions. Ultimately dietary diversity score (DDS) was computed out of maximum score of nine and categorized into low (< 4), medium (4–5) or high (> 5) [24]. Mid-upper arm circumference (MUAC) and height was measured following standard procedures. Pregnant women with MUAC less than 21 cm were considered as malnourished [25].

Stool samples collected as part of the routine ANC are used for the study. Stool samples were collected from the women using clean screw-top containers and within an hour of collection stool microscopy was made at the respective health centers by five Bachelor’s degree holder laboratory technicians. Direct microscopy with Formalin-ether concentration technique was employed to identify the parasites [26]. In order to reduce inter-observer variation, stool samples were independently examined by two technicians.

Hemoglobin concentration was determined from a random finger-tip capillary blood using HemoCue 301. Hemoglobin measurements were adjusted for altitude according to the recommendation of the WHO [27] and, classified as normal (> 11.0 g/dl) or mild (10.0–10.9 g/dl), moderate (7.0–9.9 g/dl) and severe (< 7.0 g.dl) anemia.

This manuscript is organized according to the STrengthening the Reporting of OBservational studies in Epidemiology (STROBE) guideline and the STROBE checklist is provided as a supporting file (Additional file 1).

### Data management and analysis

Collected data were checked for completeness and consistency and entered into SPSS version 20 statistical package for analysis. Data were described using frequency distributions, tables and measures of central tendency and dispersion. The association between specific type of intestinal parasites and anemia was presented using chi-square or fisher exact test. Further, the relationship between intestinal parasitosis and anemia was determined using bivariable and multivariable binary logistic regression analyses and the outputs are presented using crude (COR) and adjusted (AOR) odds ratio. Independent variables having *p*-value of 0.2 or less in the bivariable models were considered as potential confounders, and hence adjusted in the multivariable model. The thirteen variables that were adjusted in the multivariable model were age, educational status, place of residence, monthly income, family size, birth interval, parity, history of deworming in the last 6 months, iron supplementation during the pregnancy, feeding pattern during pregnancy (as usual or more than usual), receiving nutritional counseling during pregnancy, MUAC and toilet ownership. In the multivariable model multicollinearity among independent variables was assessed using Variance Inflation Factor and found to be within acceptable limits (< 10). The model goodness-of-fit was evaluated using the Hosmer-Lemeshow statistic.

### Ethical consideration

Ethical clearance was obtained from Institutional Review Board of College of Medicine and Health Science, Hawassa University. Data were collected after taking informed verbal consent from each of the study participants. As all the study subjects were 18 years or above, consent was directly taken from them. Verbal consent, rather than written consent, was preferred because one-third of the study subjects did not have formal education. The same was approved by the institutional review board. Pregnant mothers found to have anemia and/or intestinal parasites were linked with clinicians at the health centers and received treatment.

## Results

### Characteristics of the respondents

A total of 349 pregnant women following ANC took part in the study making the response rate 99.1%. The participants were selected from five health centers located at altitude ranging from 1761 to 2690 m ASL. Nearly three-fourths (72.2%) of the study participants were drawn from the midland kebeles. The majority of the women (72.2%) were rural dwellers.

The mean (± SD) age of the women was 25.7(± 4.7) years and the vast majority (87.1%) were within the age bracket of 20 to 35 years. The majority of women worked as housewives (80.2%), were from the Sidama ethnic group (72.8%), were predominantly Protestant (85.4%) and nearly 1 in 3 (29.2%) were illiterate. The median monthly income was 2500 Ethiopian birr (equivalence of 90 USD) and ranged from 600 to 5995 birr. The average (± SD) household size was 4.8 (±1.8) (Table 1).

**Table 1:** Socio-demographic and economic characteristics among pregnant women attending antenatal care in health centers of Wondo Genet district, Southern Ethiopia, 2018

Variables (*n* = 349)	Frequency	Percent
Age (years)		
18–20	36	10.3
20–35	304	87.1
> 35	9	2.6
Ethnic group		
Sidama	254	72.8
Oromo	54	15.5
Amhara	19	5.4
Others	22	6.3
Religion		
Protestant	298	85.4
Muslim	33	9.5
Orthodox	18	5.2
Place of residence		
Rural	252	72.2
Urban	97	27.8
Educational status (*n* = 347)		
Illiterate	102	29.2
Informal education	35	10.0
Primary level	147	42.7
Secondary level or above	63	18.1
Education level of the husbands		
Illiterate	72	20.6
Informal education	51	14.6
Primary level	115	33.0
Secondary level and above	111	31.8
Occupation of the respondent		
Housewife	280	80.2
Merchant	39	11.2
Government employee	25	7.2
Daily laborer	5	1.4
Occupation (husband)		
Farmer	176	50.4
Merchant	105	30.1
Government employee	48	13.8
Daily laborer	20	5.7
Monthly income of the household (ETB)		
< 2480 Eth birr	166	47.6
> = 2480 Eth birr	183	52.4
Household size		
1–4 member	178	51.0
> = 5 member	171	49.0

During the study the mean gestational age of the women was 26.6 (±5.5) weeks. Near to two-thirds (63.3%) were in their third trimester and the remaining (36.7%) were in the second trimester. The median parity was 2 and ranged from 0 to 8. About 18.6 and 11.7% of the study participants were nulliparas and grand multiparas, respectively. Among those who had at least two births before, 45.0% had short (less than 2 years) birth interval.

Three fourth (78.5%) of the respondents use piped water as their usual source of drinking water; while remaining 14.6 and 6.9% use protected and unprotected well/spring, respectively. Majority (81.4%) of the women reported that they somehow treat water before consumption. The approaches used for treating water include: adding disinfectants (74.3%), strain through cloth (16.9%), use of water filter (6.7%) and boiling (2.1%). About three-fourths (71.3%) owned a toilet facility.

### Knowledge of pregnant women on anemia

Out of the 349 respondents, 76.8% reported they had ever heard of anemia before. Among those who were aware of anemia, 83.9, 96.3 and 48.1% knew anemia during pregnancy can be caused by bleeding, under-nutrition and infections, respectively. Out of total respondents, 72.8% were aware of at least one possible consequence of anemia in pregnancy. The most frequently mentioned consequences were: perinatal mortality (92.5%), maternal mortality (82.3%), low birthweight (66.1%) and neonatal morbidity and mortality (55.5%). Three-fourths of the respondents (74.5%) knew anemia is a preventable condition. The most commonly cited preventive measures were: eating diversified diet (97.7%), taking iron and folic acid tablets during pregnancy (94.6%), birth spacing (88.5%), preventing infection including intestinal parasites (47.7%) and specifically taking deworming medications (43.0%).

### Dietary diversity and nutritional status of pregnant women

The overall quality of diet of the pregnant women was also assessed based on the diet consumed in the preceding day. Cereals (99.1%), vitamin A rich fruits and vegetables (88.3%), roots and tubers (86.2%) and foods made of oil, fat or butter (83.1%) were consumed by the vast majority of the subjects. Legumes (69.6%) and milk or milk products (55.3%) were also taken by more than half of them. Food groups that were less frequently consumed were: other fruits (37.0%), other vegetables (23.5%), eggs (18.1%), flesh foods (12.6%) and fish (0.0%).

The mean (± SD) DDS was 4.0 (± 1.6). Only 19.5% consumed from 6 or more groups indicative of high dietary diversity. Conversely, 38.4% had medium (4–5 food groups) and 42.1% had low DDS (less than 4 food groups).

The mean MUAC of the respondents’ was 22.8 (±1.9) cm. About one-fifth (20.6%) of the pregnant women had MUAC less than 21 cm suggestive of acute malnutrition. Smaller proportions (1.1%) of the pregnant women had short stature (height less than 145 cm).

Fifty five percent of the pregnant women took deworming medication at least once in the preceding 6 months of the survey. Similarly, 45.3% reported that they took iron supplement during the current pregnancy.

### Prevalence of intestine parasitosis and anemia

The overall prevalence of intestinal parasite infection was 38.7% (95% CI: 33.6–43.8%). One-tenth (9.7%) were infected with polyparasites. *A. lumbricoides* was the most common infection (24.9%) followed by hookworms (11.2%), *G. lamblia* (5.4%), *E. histolytica* (3.4%), *T. trichiura* (2.9%) and *S. mansoni* (2.3%).

The mean hemoglobin concentration was 12.3 (±1.9) g/dl and ranged from 7.1 to 18.2 g/dl. It was found that 31.5% (95% CI: 26.6–36.4%) pregnant women were anemic. The prevalence of mild and moderate anemia were 21.5 and 10.0%, respectively. None of the women had severe anemia.

### Association between intestinal parasitosis and anemia

The prevalence of anemia among pregnant women infected with intestinal parasite (55.6%) was significantly higher than the prevalence among women who were not infected (16.4%) (*p* <  0.001). Nearly all of the mothers infected with hookworm (92.3%) were anemic. Further the prevalence of anemia was alarmingly high among mothers diagnosed with other parasites: *G lamblia* (78.9%), *S mansoni* (75.0%), *E histolytica* (50.0%), *T trichiura* (50.0%) and *A lumbricoides* 40.2%. Statistically significant association was observed between anemia and hookworm*, G. lamblia, S. mansoni and A. lumbricoides* infections (*p* <  0.05) (Table 2).

**Table 2:** Association between specific types of intestinal parasitosis and anemia among pregnant women from Wondo Genet district, Southern Ethiopia, 2018

Type of helminth infection	Anemia		Prevalence of anemia (%)	Statistical test (*p*-value)^×^
	Yes	No		
Any helminth infection				
Yes	75	60	55.6	< 0.001^*^
No	35	179	16.4	
Hookworm				
Yes	36	3	92.3	< 0.001^*^
No	74	236	23.9	
*Giardia lamblia*				
Yes	15	4	78.9	< 0.001^*^
No	95	235	28.8	
*Schistosoma mansoni*				
Yes	6	2	75.0	0.007^*^
No	104	237	30.5	
Ascaris lumbricoides				
Yes	35	52	40.2	0.043^*^
No	75	187	28.6	
*Entamoeba histolytica*				
Yes	6	6	50.0	0.205
No	104	233	30.9	
Trichuris trichiura				
Yes	5	5	50.0	0.298
No	105	234	31.0	

× Chi-square test for the first 5 variables and fisher exact test for the last 2 variables

* Statistically significant difference at *p*-value of 0.05

The bivariable logistic regression analysis suggested women who were infected with intestinal parasites had 6 times increased odds of anemia (COR = 6.39, 95% CI: 3.89–10.50) as compared to their counterparts. In the multivariable model in which 13 potential confounders were adjusted, the odds of anemia were also six times increased (AOR = 6.14, 2.04–18.45) among individuals infected with intestinal parasites (Table 3). The outputs of the full regression model is now provided as a supporting file (Additional file 2).

**Table 3:** Association between intestinal parasitosis and anemia among pregnant women from Wondo Genet district, Southern Ethiopia, 2018

Intestinal parasitosis (*n* = 349)	Anemia		COR	AOR^a^
	Yes	No		
Yes	75	60	6.39 (3.89–10.50)^*^	6.14(2.04–18.45)^*^
No	35	179	1	1

^a^Adjusted for age, educational status, place of residence, family size, monthly income, birth interval, parity, history of deworming in the last 6 months, iron supplementation during the pregnancy, feeding pattern during pregnancy (as usual or more than usual), receiving nutritional counseling during pregnancy, MUAC and toilet ownership

*Statistical significant assocatin at *p*-value of 0.05

## Discussion

The study suggested that more than one-third (38.7%) of pregnant women in Wondo Genert have intestinal parasitic infections and co-infection with polyparasites was common. Further, 31.5% have low hemoglobin level indicative of anemia. After adjusting for potential confounders, we observed a strong positive association between intestinal parasitic infection and anemia.

The prevalence of anemia reported in this study is within the 20 to 40% range indicative of moderate public health significance of anemia in a population [2]. Multiple community- or facility-based surveys in Ethiopia concluded the same [3, 28–31]. The recent DHS found 24% of women of reproductive age and 29% of pregnant women in Ethiopia are anemic [3]. Facility-based surveys in Butajira general hospital, Southern Ethiopia (27.6%) [28]; Bisdimo district hospital, Southeast Ethiopia (27.9%) [29]; Nekemte Referral hospital, Western Ethiopia (29%) [30]; and Arba Minch town health institutions, Southern Ethiopia (32.8%) [31], also reported comparable prevalence figures.

In this study the overall magnitude of the intestinal parasitic infection was 39%, with the highest prevalence for *A. lumbricoides* (25%) and hookworms (11%). Other studies in Ethiopia reported assorted magnitudes and patterns, suggesting the existence of substantial variation in the epidemiology of intestinal parasites in the country [15, 22, 31–33]. A study in Mecha district, Northwest Ethiopia reported an exceptionally high prevalence (70%) and identified *A. lumbricoides* (33%), *S. mansoni* (17%) and Hookworms (14%) as the predominant infections [32]. A study among pregnant women following ANC in a referral hospital in Northwest Ethiopia reported a comparable figure to our finding (32%) yet the major infections were protozoan: *G. lamblia* (13%) and *E. histolytica* (8%) [33]. A similar study conducted in a district hospital in Hossana town, reported 30% total prevalence and *A. lumbricoides* was the commonest infection affecting 10% of the women [22]. A study in West Gojam zone estimated 37% aggregate prevalence and, hookworm was most frequently encountered parasite (19%) [34]. A study in Western Ethiopia based on data from multiple health centers found 25% prevalence of parasitosis with the predominance of Hookworms (15%) followed by *A. lumbricoides* (7%) [15].

After controlling for potential confounders including multiple socioeconomic status indicators, we observed a statistically significant and strong association (AOR = 6.14) between intestinal parasitosis and anemia. In Ethiopia, a couple of studies conducted in Eastern Wollega (AOR = 1.8) and Gilgil Gibe Dam area (AOR = 1.8) reported significant but weaker associations [15, 35]. The differences observed in the strength of association can possibly be due to variation in intensity of parasitic infections or other contextual factors not studied in the study. As described earlier, the observed association could be explained by several biological mechanisms. Intestinal parasites may impair iron status by sucking blood from intestinal wall or by facilitating chronic blood loss, reducing appetite and nutrient intake, competing for iron and causing diarrhea/dysentery [12, 13].

This finding may imply that, on top of the routine prenatal iron supplementation, preventive or therapeutic deworming integrated with ANC may help to reduce the burden of maternal anemia. From public health perspectives this would give more sense in consideration of the fact that merely half of the pregnant women in our study received deworming medications in the preceding 6 months. According to the national guideline of Ethiopia, pregnant women should be routinely dewormed in the second or third trimester of pregnancy. Yet, a national survey indicated that in 2016 only 6% of the pregnant women received deworming medication [3].

The strength of the study is the fact that it enrolled relatively large number of pregnant women (*n* = 349) from multiple health facilities and presented prevalence figures for various intestinal parasites. Further, we attempted to measure and accounted for multiple confounders that can independently explain the association between the variables of interest. Yet, the following shortcomings should be taken into considerations while interpreting our findings. Like many other observational studies, we adjusted for potential confounders using regression model. Yet, residual confounding or confounding from unmeasured confounding (e.g. malaria, HIV, other co-morbidities) cannot be excluded. As nearly half of the study subjects received deworming treatment in the preceding 6 months of the survey, the study is likely to underestimate the underlying magnitude of the problem. Due to the cross-sectional nature of the study we could not be able to capture the seasonal variations in the prevalence of intestinal parasitosis. Previous studies have witnessed that prevalence of many intestinal parasites in human population is subjected to inter-seasonal fluctuation [36, 37]. Further, as the study was limited to pregnant women attending ANC, the findings may not be directly extrapolated to pregnant women devoid of prenatal care. In addition, we did not measure intensity of parasitic infection and accordingly we did not manage to explore the does-effect relationship between intestinal parasitosis and anemia. It is also important to note that the study was only limited to intestinal parasitosis and did not look into the effect of malaria – an important hemoparasite in the locality.

## Conclusion

The study indicated that 39% of the pregnant women in Wondo Genet district have intestinal parasitic infections and 32% were anemic. The most common types of intestinal parasites were *A. lumbricoides* and hookworm. Co-infection with multiple parasites was commonly encountered. Further, a strong association was observed between intestinal parasitosis and anemia. Strengthening the existing water, sanitation and hygiene programs and routine deworming of pregnant mothers may help to reduce the burden of anemia.

## Additional files

Additional file 1: STROBE Statement. (DOC 86 kb)

Additional file 2: Full output of multivariable binary logistic regression analysis. Wondo Genet district, Southern Ethiopia, 2018. (DOCX 20 kb)

## Data Availability

The datasets analyzed during the current study are not publicly available due institutional regulation but are available from the corresponding author on reasonable request.

## Abbreviations

ANC: Antenatal Care
AOR: Adjusted Odds Ratio
ASL: Above Sea Level
CI: Confidence Intervals
COR: Crude Odds Ratio
DHS: Demographic and Health Survey
FAO: Food and Agriculture Organization
MUAC: Mid-upper arm circumference
OR: Odds Ratio
SD: Standard Deviation
WHO: World Health Organization
