# Supplementary material for: Prevalence of intestinal parasitic infection and its association with anemia among pregnant women in Wondo Genet district, Southern Ethiopia: a cross-sectional study
Source: BMC Infect Dis. 2019 May 30;19:483. doi: 10.1186/s12879-019-4135-8 (PMC6543579; doi:10.1186/s12879-019-4135-8)
Supplement: Supplementary file 2 — Full output of multivariable binary logistic regression analysis. Wondo Genet district, Southern Ethiopia, 2018. (DOCX 20 kb) [file 12879_2019_4135_MOESM2_ESM.docx]

| Variable (n =349) | Anemia | | COR | AOR |
| --- | --- | --- | --- | --- |
|  | Yes | No |  |  |
| Intestinal parasitosis |  |  |  |  |
| Yes | 75 | 60 | 6.39(3.89-10.50) | 6.14(2.04-18.45)** |
| No | 35 | 179 | 1 | 1 |
| Age of respondents |  |  |  |  |
| 15-25 years | 42 | 132 | 1 | 1 |
| >25 years | 68 | 107 | 1.99(1.26-3.17) | 0.74(0.22-2.44) |
| Educational status |  |  |  |  |
| Illiterate | 52 | 50 | 1 | 1 |
| Read & write | 16 | 18 | 7.28(3.15-16.8) | 2.83(0.53-15.44) |
| Primary | 34 | 115 | 6.2(2.30-16.9) | 0.64(0.19-2.16) |
| Secondary or above | 8 | 56 | 2.1 (0.89-4.76) | 1.02(0.11-9.35) |
| Place of residence |  |  |  |  |
| Urban | 18 | 79 | 1 | 1 |
| Rural | 92 | 160 | 2.52(1.42-4.47) | 2.2(0.47-10.20) |
| Monthly income level |  |  |  |  |
| < 2480 birr | 74 | 92 | 3.59(1.25-10.36) | 4.29(1.4-13.13)** |
| >2480 birr | 36 | 147 | 1 | 1 |
| Family size |  |  |  |  |
| 1-4 members | 14 | 126 | 1 | 1 |
| >= 5 members | 96 | 113 | 7.65(4.13-14.15) | 11.55(2.29-58.23)** |
| Birth interval |  |  |  |  |
| <= 2years | 53 | 75 | 1.98(1.20-3.27) | 1.19(0.44-3.30) |
| >2years | 41 | 115 | 1 | 1 |
| Parity |  |  |  |  |
| Nullipara | 16 | 49 | 1 | 1 |
| Primipara | 16 | 49 | 1.00(0.45-2.22) | 1.17(0.13-10.20) |
| Multipara | 78 | 141 | 3.55(1.54-8.16) | 0.22(0.05-1.01) |
| Feeding pattern |  |  |  |  |
| < usual or as usual | 95 | 149 | 3.79(1.09-13.15) | 2.04(0.18-23.32) |
| >usual | 15 | 90 | 1 | 1 |
| Nutrition counseling |  |  |  |  |
| Yes | 18 | 189 | 1 | 1 |
| No | 92 | 50 | 19.32(10.67-34.98) | 11.51(3.51-37.8)** |
| IFA supplemented |  |  |  |  |
| Yes | 32 | 126 | 1 | 1 |
| No | 78 | 113 | 2.72(1.68-4.41) | 1.3(0.39-4.36) |
| Dewormed in 6 months |  |  |  |  |
| Yes | 5 | 188 | 1 | 1 |
| No | 105 | 51 | 5.69(2.20-14.72) | 4.99(0.77-32.26) |
| Toilet ownership |  |  |  |  |
| Yes | 42 | 207 | 1 | 1 |
| No | 68 | 32 | 10.47(6.13-17.89) | 1.5(0.5-4.57) |
| MUAC |  |  |  |  |
| < 21 cm | 61 | 11 | 25.80(12.67-52.61) | 9.47(2.61-34.41)** |
| >= 21 cm | 49 | 228 | 1 | 1 |

Supporting file 2: Full output of multivariable binary logistic regression analysis. Wondo Genet district, Southern Ethiopia, 2018.
